# Characterization of Copy Number Variation’s Potential Role in Marek’s Disease

**DOI:** 10.3390/ijms18051020

**Published:** 2017-05-09

**Authors:** Lingyang Xu, Yanghua He, Yi Ding, Guirong Sun, Jose Adrian Carrillo, Yaokun Li, Mona M. Ghaly, Li Ma, Huanmin Zhang, George E. Liu, Jiuzhou Song

**Affiliations:** 1 Department of Animal & Avian Sciences, University of Maryland, College Park, MD 20742, USA; xulingyang@163.com (L.X.); yhe12345@umd.edu (Y.H.); dingyi.umd@gmail.com (Y.D.); grsun2000@126.com (G.S.); carrillo@umd.edu (J.A.C.); liyaokun1986@163.com (Y.L.); monaghaly19@yahoo.com (M.M.G.); lima@umd.edu (L.M.); 2 Animal Genomics and Improvement Laboratory, USDA-ARS, Beltsville, MD 20705, USA; 3 Institute of Animal Science, Chinese Academy of Agricultural Sciences, Beijing 100864, China; 4 College of Animal Science and Veterinary Medicine, Henan Agricultural University, Henan Innovative Engineering Research Center of Poultry Germplasm Resource, No. 95 Wenhua Road, Zhengzhou 450002, China; 5 College of Animal Science and Technology, Northwest A & F University, Yangling 712100, China; 6 Department of Animal Production, Faculty of Agriculture, Cairo University, Giza, Egypt; 7 USDA, Agriculture Research Service, Avian Disease and Oncology Laboratory, East Lansing, MI 48823, USA

**Keywords:** copy number variation, chicken, inbred lines, recombinant congenic strains (RCS), Marek’s disease, RNA-sequencing

## Abstract

Marek’s Disease (MD) is a highly contagious pathogenic and oncogenic disease primarily affecting chickens. Chicken Lines 63 and 72, as well as their recombinant congenic strains (RCS) with varied susceptibility to MD, are ideal models to study the complex mechanisms of genetic resistance to MD. In this study, we investigated copy number variation (CNV) in these inbred chicken lines using the Affymetrix Axiom HD 600 K SNP genotyping array. We detected 393 CNV segments across all ten chicken lines, of which 12 CNVs were specifically identified in Line 72. We then assessed genetic structure based on CNV and observed markedly different patterns. Finally, we validated two deletion events in Line 72 and correlated them with genes expression using qPCR and RNA-seq, respectively. Our combined results indicated that these two CNV deletions were likely to contribute to MD susceptibility.

## 1. Introduction

High-quality poultry products are an invaluable food resource for humans. Marek’s disease (MD) causes substantial revenue loss in the global poultry industry. MD is caused by Marek’s disease virus (MDV), which is a serious problem threatening the poultry industry worldwide [1]. Currently, the control of MD mainly relies on vaccination. However, the vaccination efficacy has been experiencing erosion due to new emerging strains of MDV with escalated virulence [2], and control measure expenses. Therefore, understanding of genetic basis of MD and improving MD resistance in chickens are of great interest for the poultry industry and animal welfare.

Genetic variations and gene expression play important roles in phenotypic diversity [3,4]. Some of these variations may underlie major mechanisms that account for variations in disease resistance. Thus, the identification of these variations and potential genetic markers is important for better understanding of disease resistance, as well as for genomic predication and marker-assisted selection. Since host resistance to MD was first reported in the 1930s [5], genetic bases for resistance to MD have been extensively studied [6,7,8,9,10]. During the process, two chicken inbred lines, Line 63 and Line 72, their F1 reciprocal crosses, and the recombinant congenic strains (RCS) were developed and have been maintained at the USDA-ARS. The RCS developments, genetic and phenotypic variations in MD resistance were described previously by Bacon et al. and Chang et al. [11,12]. Briefly, Line 63 and Line 72 were crossed to generate F1; F1 hybrids were backcrossed to Line 63 to create the first backcrossed hybrid (BC_1_); BC_1_ chickens were then crossed back to Line 63 again to produce the BC_2_. Each RCS was initiated with progeny of the BC_2_ hybrids, which were reproduced under a strict scheme of full-sib mating. In the end, Line 63 is resistant to MD while Line 72 is highly susceptible to MD; and the RCS vary in MD resistance. Additionally, all of the lines of chickens share a common major histocompatibility complex B haplotype, B*2 [11].

There have been nearly 20 quantitative trait loci (QTL) regions reported to be associated with MD resistance in chickens [13,14,15]. However, none of them have been used in practical breeding for MD resistance, and the possible reasons include the highly complex nature of MD [16] and the low resolution of the QTL map [17,18]. Subsequently, the identification of candidate genes within QTLs has been attempted by utilizing comparative mapping approaches, but some drawbacks have still seriously crippled such efforts due to insufficient knowledge of the functional genes [19].

There are several types of genetic variations. In addition to single nucleotide polymorphism (SNP), copy number variation (CNV) is one of the important genetic variants. They not only affect gene expression levels, but also result in phenotypic variations, often impacting on disease susceptibility [20,21,22,23]. In domestic dogs, a CNV in *KITLG* likely causes a risk for canine squamous cell carcinoma [24]. A CNV located upstream of *HAS2* more likely causes a “wrinkled” skin in Chinese Shar-Pei Dogs [25]. Additionally, *ABCC4* copy number variation is involved in susceptibility to esophageal squamous cell carcinoma in human [26]. In farm animals, many CNV analyses have been reported [27,28,29]. Notably, many evidences have shown that CNV can contribute to complex pigmentation in chickens [30]. For example, a duplication in intron 1 of *SOX5* causes the pea-comb phenotype [31]; a deletion upstream of *SOX10* leads to the dark brown plumage color [32]. A partial duplication of *PRLR* and *SPEF2* genes is involved in late feathering [33].

As for MD resistance, the genetic characterizations of the disease have been explored by investigating differential RNA transcripts [34], the QTL mapping [13,14,15], and other integrated methods over the past years [35]. CNVs related to MD resistance were reported before using array CGH and next-generation sequencing [36,37]. However, most of them were reported in only parental lines, i.e., MD-resistant Line 63 and susceptible Line 72. Thus, it is still not well understood whether CNVs contribute to susceptibility to MD in chickens. In this study, using the Affymetrix Axiom HD 600 K genotyping array (Santa Clara, CA, USA), we performed a CNV analysis in chickens from different genetic lines, including the two highly-inbred lines, F1 reciprocal crosses of the two inbred lines, and six RCS, which significantly vary in genetic resistance to MD. By assessing genetic structures using CNV segments, we found distinct CNV distribution patterns among the chicken groups. Our results from the gene expression also confirmed that these CNVs can potentially contribute to MD in chickens.

## 2. Results

### 2.1. Identification of CNV Segments

**Figure 1:**
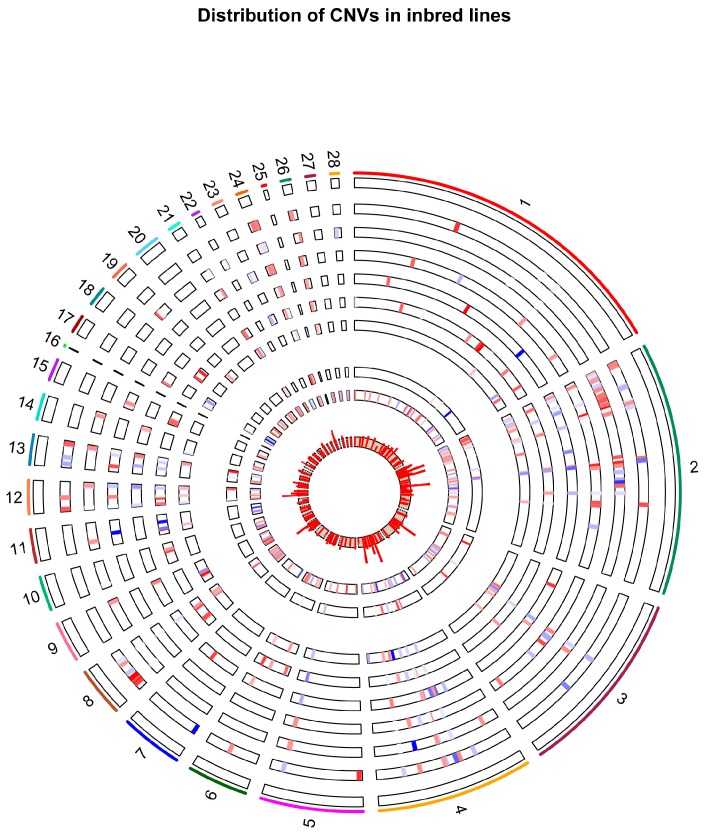
Circos plot illustrating CNV regions in eight inbred chicken lines (two F1 were not included). Regions with copy number events are plotted within the gray inner circle. Copy number changes indicated by segment mean log_2_ Ratio values are shown in the inner circle plot using the RCircos.Heatmap.Plot function in RCircos package. The outermost circle displays the chicken chromosomes (chrZ and chrW were excluded). The circles from inside to outside represent Lines 72, 63, RCS C, J, M, N, S and X. The bin heights in the inner circle represent the frequencies of CNV segments.

Using the multivariate method in SVS (SNP Variation & Suite software by Golden Helix Inc., Bozeman, MT, USA at http://goldenhelix.com/products/SNP_Variation/index.html), we detected 11,790 distinct segments across all of the inbred chicken lines. After filtering away segments with mean value between −0.3 and 0.3, we obtained 393 non-redundant segments. After removing 223 neutral segments, we finally obtained 170 CNVs. These CNVs cover a total of 831,808 bp in length and encompass 632 SNPs. On average, each CNV is about 4893 bp in length; and each contains about 3.7 SNPs. Detailed statistics are given in supplementary Table S1. In addition, we observed the highest loss frequencies (50%) on chromosomes 2, 3, and 4, while the lowest frequencies were ~3% on chromosomes 1, 8, 10, 15, 20, 21, 22, and 24 (Figure 1).

### 2.2. Gene Annotation of Chicken CNV Segments

We obtained 32 RefSeq genes within the regions of 170 CNV segments and then carried out the PANTHER classification analysis based on these genes. We found most of them were involved in cellular processes (37.9%) and metabolic processes (31.0%). However, due to the small number of genes, only one term “unclassified” was found to be significant after false discovery rate (FDR) correction. Additionally, we performed the network analysis using IPA. The identified genes were mapped to two genetic networks using IPA Core analysis. The first network, having an IPA network score of 38 and 15 focus genes, presented functions related to carbohydrate metabolism, lipid metabolism, and small molecule biochemistry. The second one, with a score of 26 and 12 focus genes, centered on nervous system development and function, organism development, and skeletal and muscular system development and function.

### 2.3. Cluster and PCA

**Figure 2:**
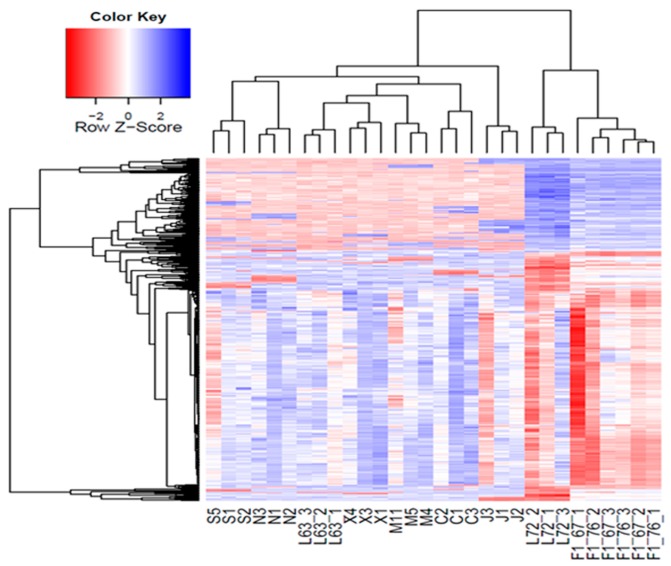
Ten chicken lines were grouped into different clusters in this heatmap plot based on CNV segment mean values (the average log_2_ Ratio values). These results revealed three samples from the same lines were always clustered together in the same branches. As expected, F1 individuals branched in an intermediary position between Line 72 and Line 63.

**Figure 3:**
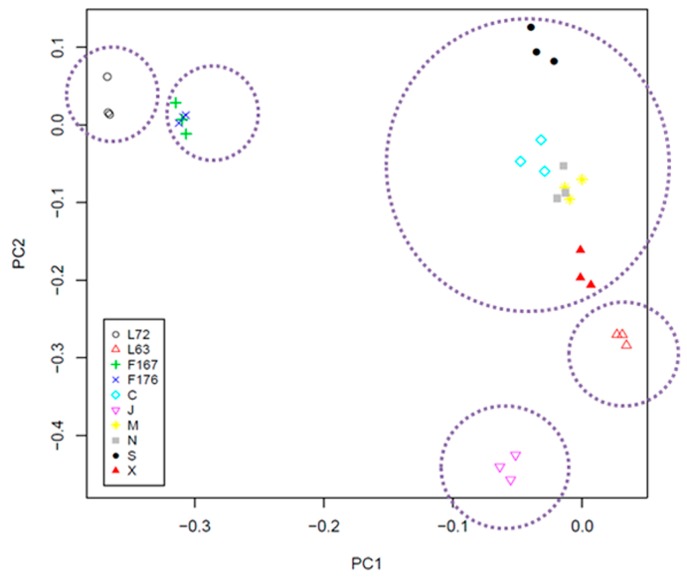
PCA plot based on the first two principal components in the ten inbred lines. These ten lines were clustered to approximate five groups, as indicated by dashed circles, which was consistent with their clustering results.

We then generated CNV maps and detected diverse CNV distributions across eight inbred chicken lines (Figure 1). Based on the mean LRR values of all CNV segments, we performed a cluster analysis and plotted a heatmap to study a global landscape of CNV, which displayed differential patterns (Figure 2). We also observed remarkably distinct patterns of stratification across these ten lines. Our results revealed three samples from the same lines were always clustered together in the same branches. As expected, F1 individuals branched in an intermediary position between Line 72 and Line 63. To investigate genetic structure in 10 inbred lines using CNVs segments, we performed a principal component analysis (PCA) using these CNV segments. Ten principal components were calculated and the first two PCs were used in the plot (Figure 3). The first PC separated the 30 chickens into two clusters, (1) Line 72 (susceptible) and F1 (hybrid F1 generation with various levels of susceptibility) and, (2) Line 63 (resistant) and other RCS lines. The ten lines were clustered to approximately five groups, as indicated by dashed circles (Figure 3), which was consistent with their clustering results. When we looked at the second PC, six RCS lines were well separated with RCS-J dispersing further away from other RCS lines and Line 63. Only RCS-C was mixed together with RCS-M and N.

### 2.4. Shared Versus Lineage-Specific Events

To investigate how frequently CNV events were lineage-specific or shared among lines, we calculated the CNV numbers shared among the five groups, as indicated by the five dashed circles in Figure 3. These five groups included Line 72, Line 63, Line J, F1s, and a group merging RCS-C, M, N, and S. Approximately 17 CNVs were detected in all the groups, which represented the commonly shared CNVs. In contrast, 12 of 170 CNVs exhibited copy number change only in Line 72, as compared to other lines (Table 1). These Line 72 lineage-specific CNVs could potentially offer certain clues to explore the genetic mechanisms of MD susceptibility.

### 2.5. Candidate CNVs Conferring Marek’s Disease

Based on the Gallus_gallus_4.0 reference genome, we further annotated the detected CNVs. We identified a CNV region encompasses *BFSP1* (beaded filament structural protein1) at chr3:11,362,776–11,381,424. The gene *BFSP1* is a major cytoskeletal element of the eye lens [38]. The abnormal function of *BFSP1* may cause diseases, such as cataracts and cortical, juvenile-onset, and nodular basal cell carcinoma [39]. Upstream of the CNV region (chr3:52,027,363–52,029,235), the *GPR126* gene (chr3:52,148,761–52,255,579) is located there, encoding the G-protein-coupled receptor 126 [40,41]. Near the CNV (chr19:3,266,850–3,271,865), there is an *ATP2A2* gene (chr19:3,274,476–3,308,472), which is correlated with different cancer types [42,43,44]. Upstream of the CNV (chr8:4,018,141–4,022,302), we found the *MTA1* gene (chr8:3,995,484–4,061,917), which is a family member of ubiquitously expressed chromatin, an integral component of the nucleosome remodeling and histone deacetylation (*NuRD*) complexes [45]. It was found that *MTA1* acts as a mandatory modifier of breast-to-lung metastasis. The significance of the normal level of *MTA1* is important because it acts as an oncogene when over-expressed [46].

**Figure 4:**
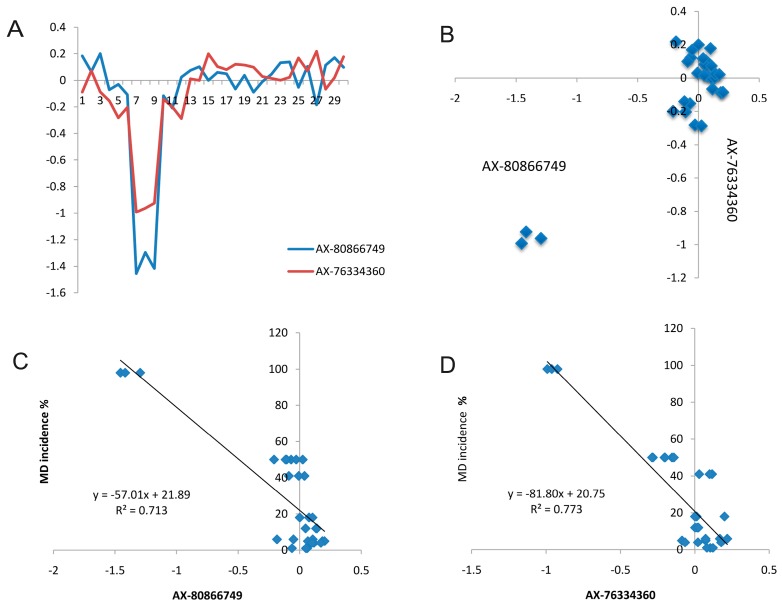
(**A**) Distribution of mean log_2_ Ratios of two deletions within 30 individuals of 10 inbred chickens lines. X-axis: individual sample number (Number 7, 8 and 9 are Line 72 individuals), Y-axis: mean log_2_ Ratio; (**B**) Scatter plot of the mean log_2_ Ratios for the same two deletions shows significant correlation between them; (**C**) Linear regression between the average MD incidences for each line and average marker intensities for CNV1 (deletion at AX-80866749); and (**D**) Linear regression between the average MD incidences for each line and average marker intensities for CNV2 (deletion at AX-76334360).

We then focused on two lineage-specific CNVs from Line 72 because their segregating patterns suggest their potential contribution to MD. These two CNVs (CNV1 and CNV2) were found on chr10: 5,716,844–5,717,897 (1054 bp in length) and chr26: 1,927,477–1,931,663 (4187 bp in length). When we checked the mean log_2_ Ratios of these two deletions (indicated by AX-80866749 and AX-76334360) across all chickens, we found all three Line 72 chickens displayed a loss or deletion pattern (Figure 4A). We then plotted the mean log_2_ Ratios of these two deletions and observed that all three Line 72 chickens can be separated clearly from the other individuals, which suggested log_2_ Ratios of the two deletions are the most important principal components with distinct differences among lines (Figure 4B). Using log_2_ Ratio from these two deletions, we also found there were significant correlations between two deletions and average MD incidences for each line with the *R*^2^ = 0.73 for deletion AX-80866749 and *R*^2^ = 0.77 for deletion AX-76334360 (Figure 4C,D).

For CNV1 (chr10:5,716,844–5,717,897), we found *TARSL2*, which is related to threonine-tRNA ligase activity, and two downstream protein-coding genes *TM2D3*, which functions in cell death or proliferation signal cascades and gene *ADAL* involving in regulating metabolism of nucleotides. The CNV2 (chr26: 1,927,477–1,931,663) contains gene *CNTN2*, which synthesizes neuronal cell adhesion molecule and contactin 2 [47]. Notably, a key role of the *CNTN2* was reported in neuronal excitability [48]. The CNV2 also contains *TAG-1* (transiently expressed axonal glycoprotein 1), which functionally involves in the formation of axon connections in the developing nervous system and glial tumorigenesis. Several genes in or near CNV regions, including *CNTN2*, *DBX1*, *ATP2A2*, *BFSP1*, and others, which are involved in the function of neuronal fates and tumorigenesis. Considering the symptoms known to occur after infection with Marek’s disease, we suspected that CNV in these regions could likely be involved in the susceptibility and resistance of Marek’s disease.

### 2.6. Validation of CNVs

**Figure 5:**
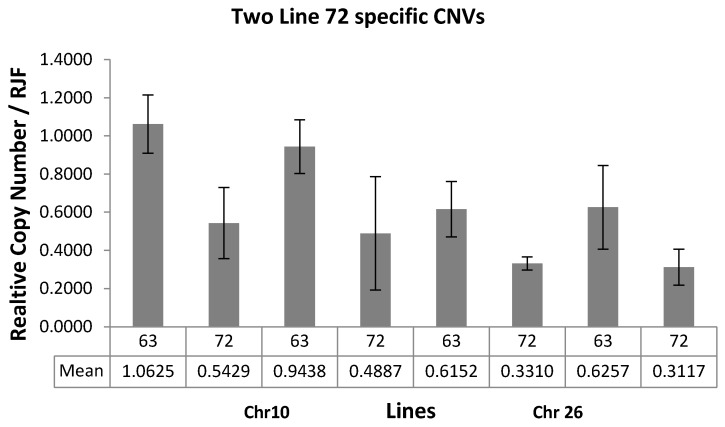
Quantitative PCR was performed on two Line 72 lineage-specific deletion CNVs (chr10:5,716,844–571,784,053 and chr26:1,927,477–1,931,663) using two independent primers per CNV.

We performed a qPCR validation on two Line 72 lineage-specific deletion CNVs: chr10:5,716,844–5,717,897 and chr26:1,927,477–1,931,663, using two independent primers per CNV (Table S2). On chr10, most of Line 63 had a similar copy number, while Line 72 had about 50% decreased copy numbers when compared with RJF. On chr26, both Line 63 and Line 72 displayed decreased copy numbers with Line 63 showing half of the copy numbers and Line 72 showing one third of RJF’s copy numbers (Figure 5). Therefore, the copy numbers of these two loci were found significantly lower in Line 72 as compared to Line 63, again supporting that our array-based deletion calls and suggesting that they are potentially linked to MD susceptibility as suspected above.

### 2.7. Influence of Deletions on Local Gene Expression

To test the correlation between deletions and expression levels of nearby genes, we extracted genomic regions within ±500 kb of each deletion. For CNV1’s extended region on chr10, spanning 1,080,805 bp (chr10:5,146,745–6,227,549), we observed several genes, including *MCEE*, *TARSL2*, *TM2D3*, *ADAL*, and *CHRNA7*. This region also overlapped with two QTL regions, including FP:2225, involving a feather pecking trait [49] and SPLWT:2224, related to spleen mass [50]. In spleen, we observed three transcripts, ENSGALG00000026271, ENSGALG00000004038, and ENSGALG00000004046 downstream of the deletion (ENSGALG00000003985) at chr10 were upregulated in Line 63 compared to Line 72. These regions contain genes *TM2D3*, *ADAL*, and Ensembl gene *LARP6* (Table S3), however, only *TM2D3* remained significant after FDR correction. While in thymus, we identified one upregulated transcript and two upregulated transcripts upstream and downstream of CNV1 in Line 72, including Ensembl gene *TJP1* (Table S3), but none of them were significant after FDR correction.

For CNV2 at chr26, spanning 485,704 bp (chr26:1,676,675–2,162,378), we also observed two overlapped QTL regions. They are BURSA:2384 involving the Bursa of Fabricius size trait [50], and THYMWT:21817, which are identified as being related to thymus weight. This region contains genes *NFASC*, *CNTN2*, *RBBP5*, and *DSTYK*. In spleen, we found no significant change of gene expression around deletion (embedded in ENSGALG00000000653) (Table S3). However, we identified two transcripts which were upregulated in Line 63 downstream of the deletion, responding to genes *DSTYK* and Ensembl gene *TMCC2* (Table S3), but again none of them were significant after FDR correction.

## 3. Discussion

Copy number variations in the chicken genome have been explored before by many groups in the past years. Most of these previous studies focused on the CNV discoveries using low-density SNP and CGH arrays [36,51,52,53,54,55,56]. A recently-published study has identified 3154 CNVs, grouped into 1556 CNV regions, using an Agilent 244 K chicken array in a wide variety of chicken breeds [57]. Another study carried out a genome-wide CNV analysis in two broiler lines divergently selected for abdominal fat content using an Illumina chicken 60 K SNP chip [58]. There were also some reports about CNV studies in inbred chicken lines using array CGH [36]. Additionally, a recent study has published in ten Italian chicken lines to study the population genetics based on SNP and CNV [59]. One of the main difficulties is that, unlike simple deletions and duplications, efficient genotyping tools with high accuracy are still lacking for complex CNVs (for example, multi-allelic CNVs) [60]. Here we utilized the SVS multivariate CNV calling algorithm because this method can facilitate downstream CNV association studies. To our knowledge, this is one of the first detailed studies in chickens to investigate CNV across closed-related chicken populations using a high-density chicken SNP array (600 K). In total, we found 66 out of 170 CNVs overlapped with MD-related quantitative trait loci (QTL) regions. Our results also revealed additional novel genetic variations underlying MD than those revealed by SNPs and microsatellites alone. The new alternative frameworks offered in our studies will help to explain the missing heritability of complex traits in chickens and other farm animals [22].

Previous studies have identified chicken CNV using low-density platforms with limited sample sizes in different chicken lines. It is difficult to make comparisons across all of these studies. Thus, we only performed comparisons with the recent CNV results reported in chickens. We found 18 CNV (with a total of ~2 Mb), which overlapped with the large-scale CNV analysis in chickens [57]. We also compared CNV discovered in Line 63 and Line 72 in this study with our earlier results using a CGH array [36]. We found no overlapping CNVs, which may be, in part, related to different array platforms and analysis methods. We also compared with the CNV identified in Lines 63 and 72 recently using next generation sequencing [37], and found approximately 50 CNVR overlaps. Notably, our study mainly focused on the common CNV detection using the HD SNP array, which was different from the CNV screen using next generation sequencing. Further, our study explored the genetic structure based on CNV in inbred chickens. The PCA analysis showed clearly that the first two PCs can separate all chickens into unique groups. This result is similar to the SNP-based PCA result (Xu et al., 2017, described in a separate manuscript). A recent study suggested that, like SNPs, CNV can separate Italian chicken populations similarly well [59]. Compared to their study, our study further confirms that CNV markers can be used to study the genetic variability in diverse chicken lines. Thus, similar to what have been observed in zebrafish [61], our results revealed direct evidences that CNVs can be used to characterize individuals based on their unique genetic structures, which could possibly contribute to lineage-specific phenotypes.

Similar to mouse and dog models, inbred chicken lines and recombinant congenic strains are also ideal model systems to investigate the mechanisms that underlie complex disease traits. In the current study, we investigated CNVs among diverse inbred lines and found 12 unique CNV segments in Line 72, which could be associated with MD. Importantly, we found two CNVs, which were deletions in all three chickens from Line 72 and can separate them clearly into a distinct cluster. Remarkably, there were significant correlations between the log_2_ Ratio (i.e., CNV status) and average MD incidence for both deletions. Since copy number variations are associated with gene expression [4,62,63], using RNA sequencing in lymphoid tissues (spleen and thymus), we found some differentially-expressed genes between Line 63 and Line 72 near the CNVs. Like other species [64,65], our results suggest the CNVs could affect the expression of nearby genes. We found a deletion on chr10 that was associated with a higher RNA level of *TM2D3* in Line 63 in the spleen. The gene regulations around two deletions are not consistent between the spleen and thymus. We speculated the regulation could associate the different epigenetic effects, such as histone modifications in various tissues [66,67].

In summary, we investigated copy number variation in inbred chicken lines using a high-density SNP array. We discovered 12 unique CNVs in Line 72, two of which were shown to affect the expression of nearby genes, providing new insights into the mechanisms of Marek’s disease.

## 4. Materials and Methods

### 4.1. Experimental Animals

A total of 30 individuals were used in the study, including three chickens from each of Line 63, Line 72, their two F1 reciprocal crosses, and six RCS. All of them were developed and have been maintained at the USDA, Agriculture Research Service, Avian Disease and Oncology Laboratory (ADOL), East Lansing, Michigan, USA. The six RCS that were sampled for this study were RCS-C, J, M, N, S, and X. All animals used in this experiment were handled closely following the Animal Care and Use Guidelines developed and approved by the Animal Care and Use Committee at ADOL (revised April 2005, Project Number 6040-31320-009-00-D) and the Guide for the Care and Use of Laboratory Animals published by the Institute for Laboratory Animal Research (Eighth Edition) in 1996.

### 4.2. Phenotypic and MD Incidence

Genetic resistance to MD in chickens is commonly evaluated with MD incidence post-MDV challenge, i.e., a percentage of birds developed MD following an exposure to MDV within each line as a treatment group. Therefore, it is an average measurement for each line, not for each individual animal (Figure S1). Based on these average MD incidences within a group, Line 63 is relatively resistant to MD, while Line 72 is highly susceptible, and the RCS vary in MD resistance [10,11,12,68,69,70,71].

### 4.3. Copy Number Variation Analysis

The CNV analysis was performed using Golden Helix SNP & Variation Suite (SVS) 8.0 (Golden Helix Inc., Bozeman, MT, USA) on data generated by Affymetrix Axiom HD 600 K genotyping array (Santa Clara, CA, USA). We first used Affymetrix Power Tools (APT) to retrieve the summarized signal intensity files. Then Log_2_ Ratio was calculated using the method supplied by APT and imported into Golden Helix SVS 8.0. A total of 580,961 SNPs were mapped on the chicken genome (assembly Gallus_gallus_4.0) to determine their positions within each autosome [72]. To normalize the log_2_ Ratios, we used the default GC correlation file (GC Reference Gallus_gallus_4.0.gc_digest.dsf) to correct for the waviness contributed by the GC content. We utilized the copy number analysis module (CNAM) under the multivariate segmenting option to segment chromosomes with a maximum of 20 segments per window, a minimum of three markers per segment, and a significance level (*p*-value) of 0.01 for pairwise permutations (*n* = 1000). CNV genotyping across individuals was according to a three-state copy number model with two thresholds at −0.3 and 0.3. To summarize the results of CNV segments, CNV regions (CNVRs) were then determined by aggregating overlapping CNVs across all samples. The gene annotation of CNVR was based on the Gallus_gallus_4.0 reference assembly from the UCSC genome browser.

### 4.4. Validation of CNVs by Quantitative Real-Time Polymerase Chain Reaction (qPCR)

To experimentally validate selected individual CNV calls, we performed qPCR on two selected deletion CNV events: CNV1 on chr10: 5,716,844–5,717,897 and CNV2 on chr26: 1,927,477–1,931,663. Primers were designed using Primer3.0 online (http://frodo.wi.mit.edu/). We used a modified ^ΔΔ*C*t^ method, as previously described [73,74,75]. For each chicken line, at least three individuals were used to do the validation. The Red Jungle Fowl (RJF) DNA was used as the reference sample. The single-copy gene VIM, i.e., vimentin [76] was used to normalize the amount of input DNA. The *C*t value of each tested sample was normalized to the reference gene first, and then the Δ*C*_t_ value was calculated between the test sample and the reference sample (RJF). The relative copy number was calculated as 2^(1−ΔΔ*C*t)^ by assuming that there are two copies in the reference region.

### 4.5. PATHER Classification and IPA Network Analysis

To study genes overlapping with CNVR, gene ontology categories were analyzed using the PANTHER (Protein ANalysis THrough Evolutionary Relationships) classification system (http://www.pantherdb.org/) as described previously [77]. The *p*-values were calculated according to the binomial test integrated in the PANTHER online tool. Bonferroni’s correction for multiple testing was applied, followed by assessment of the GO significance at a *p*-value cutoff level of 0.05. In addition, genes overlapping with CNVR were further analyzed using Ingenuity Pathways Analysis (IPA) v9.0 (Ingenuity Systems, Redwood City, CA, USA). The accessions of unique genes were imported into the software and subsequently mapped to their corresponding annotations in the Ingenuity Pathways Knowledge Base. The “Core Analysis” function included in IPA (http://www.ingenuity.com/webcite) was utilized to explore the context of networks, biological functions, and pathways. We used only human, rat, and mouse genes and all confidence levels, including experimentally observed evidence, predicted high or moderate confidence. The top significant biological functions and pathways were considered in our study.

### 4.6. Clustering and Population Genetics Analyses

Based on CNV’s mean log_2_ Ratio values, we clustered all chicken lines and then performed the principal component analysis (PCA) using prcomp function in R v13.1.

### 4.7. RNA Extraction, RNA-Sequencing, and Differential Expression Analysis

Total RNA of spleen and thymus from Line 63 and Line 72 was extracted using RNeasy Mini Kit (Qiagen, Valencia, CA, USA) and mRNA isolation was performed from total RNA using Oligotex mRNA Mini Kit (Qiagen, Valencia, CA, USA) according to the manufacture’s instruction. Approximately 300 ng of mRNA was used to synthesize the first and second strands of cDNA by SuperScriptTM III Reverse Transcriptase (Invitrogen, Waltham, MA, USA) and Second Strand cDNA synthesis kits (NEB, Ipswich, MA, USA). After purification, double-strand cDNA (dscDNA) was sonicated by a Bioruptor Sonicator (Diagenode, Denville, NJ, USA). Two biological replicates were used in the RNA-Seq library construction and RNA sequencing was conducted on an Illumina Hiseq 2000 (Illumina, San Diego, CA, USA) using 50 bp single end reads (SE50). Raw sequenced data were checked for quality considering the read counts, overall read quality, and read distribution. Then we aligned clean reads onto Gallus_gallus-4.0 using Bowtie version 1.0.0 [78]. In order to control the mapping quality, the first 15 bps from the original 50 bp of reads were trimmed. The counting of reads per gene was executed by the *summarizeOverlaps* function implemented in R v3.0.2. After dispersion estimation, we used the R package *edgeR* and corresponding complementary functions GLM to analyze the count reads data and perform a comparison analysis. Differentially-expressed genes (DEG) were filtered by a threshold of 0.05 after FDR adjustment [79].

## Figures and Tables

**Table 1:** Twelve specific CNV segments in Line 72 as compared to other lines.

Chr	Start	End	Length	Genes
1	84,250,750	84,254,670	3920	*TBC1D23*
2	69,574,893	69,579,418	4525	*CDH6*
2	74,099,026	74,100,933	1907	
2	81,592,992	81,594,465	1473	*GRB10*
2	115,475,473	115,484,664	9191	*CPA6*
2	116,094,599	116,097,298	2699	*COPS5*
3	43,999,153	44,003,340	4187	
3	53,309,153	53,311,383	2230	*TXLNB*
4	88,905,580	88,906,840	1260	*GFRA4*
10	5,716,844	5,717,897	1053	*TARSL2*
17	7,100,919	7,102,218	1299	*VAV2*
26	1,927,477	1,931,663	4186	*CNTN2*

## Supplementary Materials

Supplementary materials can be found at www.mdpi.com/1422-0067/18/5/1020/s1.
