# Supplementary material for: Characterization of Copy Number Variation’s Potential Role in Marek’s Disease
Source: Int J Mol Sci. 2017 May 9;18(5):1020. doi: 10.3390/ijms18051020 (PMC5454933; doi:10.3390/ijms18051020)
Supplement: Supplementary file 1 [file ijms-18-01020-s001.zip › Figure S1.pdf]

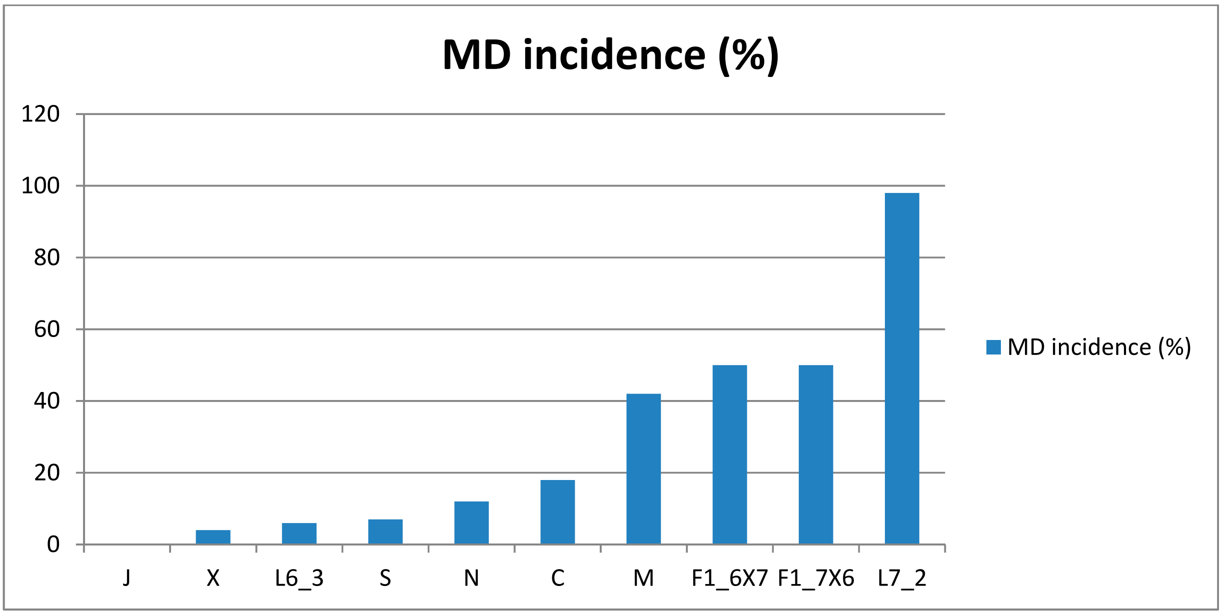

Figure S1. Histogram plot of MD incidence (%) rate. MD resistance in chickens is generally evaluated with MD incidence (induced gross tumors by MDV) and survival days post MDV challenge. The MD incidence data presented in Figure S1 were totally based on numbers of chickens that developed tumors post MDV challenge. The calculation was done as the ratio of number of birds with tumors/total number of birds challenged within each of the lines multiplied by 100.
